# Supplementary material for: Cross-cutting scenarios and strategies for designing decarbonization pathways in the transport sector toward carbon neutrality
Source: Nat Commun. 2022 Jun 24;13:3629. doi: 10.1038/s41467-022-31354-9 (PMC9232649; doi:10.1038/s41467-022-31354-9)
Supplement: Supplementary file 1 — Supplementary Information [file 41467_2022_31354_MOESM1_ESM.pdf]

Supplementary information for

Cross-cutting scenarios and strategies for designing decarbonization pathways in the transport sector toward carbon neutrality

Runsen Zhang [1, 2, *], Tatsuya Hanaoka [2]

1. Graduate School of Advanced Science and Engineering, Hiroshima University, 1-5-1 Kagamiyama, Higashihiroshima 7398529, Japan

2. Social Systems Division, National Institute for Environmental Studies, Tsukuba 3058506, Japan

*Corresponding author: rzhang@hiroshima-u.ac.jp

Supplementary Table 1. Transport mode and technology options.

Category	Technology options
Car	Gasoline cars (emission standards 1-6), gasoline hybrid electric cars, gasoline plug-in hybrid electric cars, diesel cars (emission standards 1-6), diesel hybrid electric cars, diesel plug-in hybrid electric cars, natural gas cars, battery electric cars, fuel cell cars
Bus	Diesel buses (stock, flow, high-efficient), hybrid electric buses, natural gas buses, battery electric buses
Two-wheeler	Gasoline two-wheelers (stock, flow, high-efficient), electric two-wheelers
Passenger rail	Diesel trains (stock, flow, high-efficient), electric trains (stock, flow, high-efficient)
Small truck	Diesel trucks (emission standards 1-6), hybrid electric trucks, battery electric trucks
Large truck	Diesel trucks (emission standards 1-6), hybrid electric trucks, fuel cell trucks
Freight rail	Diesel trains (stock, flow, high-efficient), electric trains (stock, flow, high-efficient)

Supplementary Table 2. Estimation results of the panel data model. We performed a two-sided t-test and derived P values with 95% confidence intervals.

Variables	Pooled model		Fixed effects model		Random effect model	
	$TD_{passenger}$	$TD_{freight}$	$TD_{passenger}$	$TD_{freight}$	$TD_{passenger}$	$TD_{freight}$
GDPCAP	-0.097	1.589***	0.820***	0.734***	0.767***	0.830***
	(0.480)	(0.000)	(0.000)	(0.000)	(0.000)	(0.000)
ROADCAP	0.090***	0.317***	0.027	0.050*	0.028	0.113***
	(0.000)	(0.000)	(0.302)	(0.054)	(0.204)	(0.000)
LANDCAP	0.470***	0.375***	0.380***	0.146**	0.385***	0.214***
	(0.000)	(0.000)	(0.000)	(0.021)	(0.000)	(0.001)
COST	0.818***	-2.268***	-0.576***	-0.263**	-0.492 ***	-0.572***
	(0.000)	(0.000)	(0.002)	(0.048)	(0.006)	(0.000)
Constant	7.754***	-9.658***	-0.497	0.076	-0.624	-0.100
	(0.000)	(0.000)	(0.328)	(0.833)	(0.583)	(0.188)
Observations	434	434	434	434	434	434
R^2 Adjusted	0.824	0.759	0.879	0.883	0.883	0.867
F test			46.492***	91.823***		
Hausman test					10.031**	253.26***

*p<0.1; **p<0.05; ***p<0.01

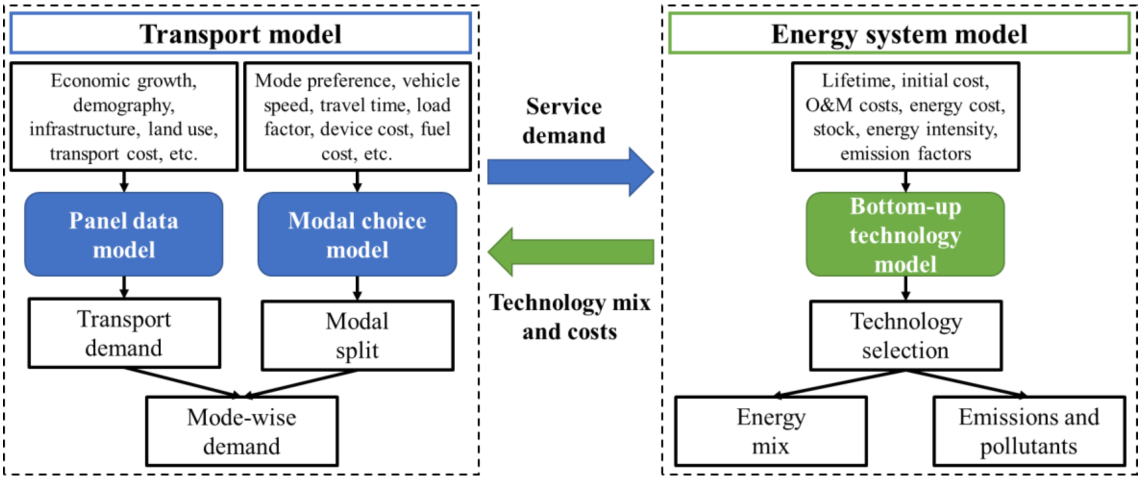

Supplementary Figure 1. Model structure.

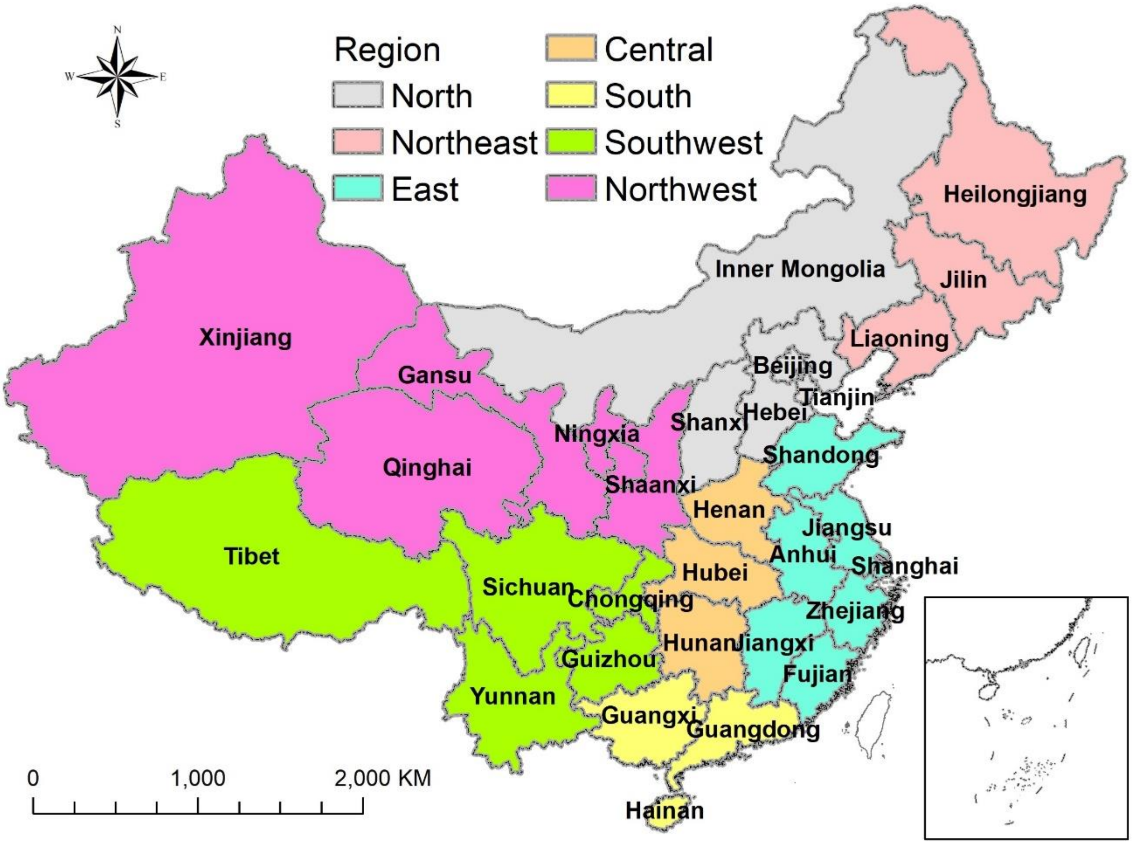

Supplementary Figure 2. The 31 provinces of mainland China.

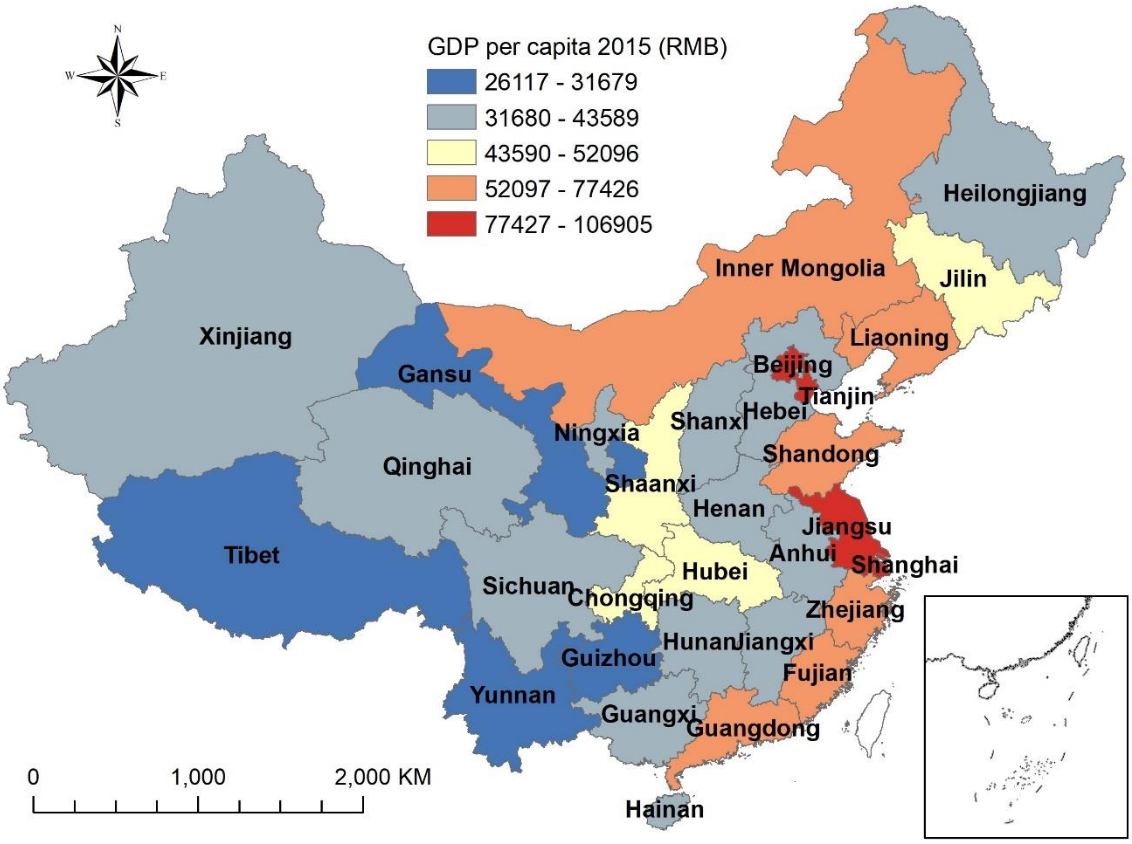

Supplementary Figure 3. GDP per capita 2015 in 31 provinces.
